# Supplementary material for: Understanding challenges and enhancing the competency of healthcare providers for disability inclusive sexual and reproductive health services in rural Nepal
Source: PLoS One. 2024 Dec 13;19(12):e0311944. doi: 10.1371/journal.pone.0311944 (PMC11642985; doi:10.1371/journal.pone.0311944)
Supplement: S1 File — (PDF) [file pone.0311944.s001.pdf]

Interview Guide for KII and FGDs

Understanding challenges and enhancing the competency of healthcare providers for disability inclusive and gender sensitive sexual reproductive health services in rural Nepal.

Interview number:
Name of the participant/s
Age:
Gender/Sexuality:
Religion:
Marital status:
Date of interview:
Venue:
Time:
Consent status:

1. What is your current role in this health institution? What kinds of services are you providing?
2. What kind of services have you provided to women with disabilities? (e.g., experience)
3. What aspects should be considered when women with disabilities come for check-ups until they leave the OPD?
4. (Probe: Whether they use Braille for communication with visually impaired women, use of symbolic language for women with reduced hearing capacity, how do you welcome them, how do you obtain consent, how do you provide medication)
5. How do you treat patients with disabilities who come alone for services?
6. What challenges do you face in providing services to women with disabilities? If there were any, how did you address them?
7. How do you communicate with individuals with hearing impairment or those who cannot speak? Whom do you question when a woman with a disability comes for services?

8. Have you received any training regarding dealing with women patient with disabilities? (e.g., Special training on how to interact with women with disabilities)
9. Do you feel the need for training on sexual and reproductive health services, orientation, and capacity strengthening for women with disabilities?
10. What special activities or arrangements are in place to facilitate the use of services for women with disabilities? If not, why not?
11. Is there a difference in the need for sexual and reproductive health services for women with disabilities compared to other women? How are they different? If not, why not?
12. In your opinion, what are the main obstacles to accessing maternal health services for women with disabilities in rural areas of Nepal? (e.g., policy, communication issues, remoteness, equipment, and transportation)
13. What changes can be made to improve access to maternal health services for women with disabilities in rural Nepal? Do you have any suggestions on how to improve the facilities for women with disabilities in rural areas of Nepal?
14. According to the data from our previous research, women with disabilities face challenges such as receiving health services from providers, discrimination, and experiencing abuse. What is your understanding of these issues? Do you think you might encounter such problems? What can be done to prevent these issues? How can a safe environment be created for them?
15. Lastly, anything you want to share?

Informed consent form

Understanding challenges and enhancing the competency of health care providers for disability inclusive and gender sensitive sexual and reproductive health services in rural Nepal

<u>Description:</u>

The research aims to delve into the barriers and facilitators in providing disability inclusive and gender sensitive sexual and reproductive health services in Karnali Province of Nepal. Furthermore, this study also seeks to understand ways to increase competency of health care providers to provide disability inclusive health services. Participants in this study are assured of confidentiality, with their personal information and identity safeguarded throughout the research process. While no remuneration is provided for participation, individuals have the autonomy to decline answering any questions that they find challenging. Voluntary participation is emphasized, offering participants the freedom to withdraw from the interview at any point. The duration of the interview is expected to range from 30 to 40 minutes, during which participants will have the opportunity to share their perspectives and experiences regarding self-care practices for SRH. Through their valuable insights, this research aims to contribute to a deeper understanding of the barriers and facilitators influencing women's engagement with self-care in the realm of sexual and reproductive health.

==

I fully understand the title, objective, scope and provide my wholehearted consent for interview and to publish the data.

Name of participant:

Signature:

Date:
