# Supplementary material for: Understanding challenges and enhancing the competency of healthcare providers for disability inclusive sexual and reproductive health services in rural Nepal
Source: PLoS One. 2024 Dec 13;19(12):e0311944. doi: 10.1371/journal.pone.0311944 (PMC11642985; doi:10.1371/journal.pone.0311944)
Supplement: Attachment — Submitted filename: Response to reviewer.docx [file pone.0311944.s002.docx]

17^th^ July 2024

**Yitagesu Habtu Aweke, Ph.D**

Academic Editor, PLOS ONE

**RE: Revision of Manuscript PONE-D-24-16726**

Dear Editor,

We would like to thank you and the reviewers for your time and providing constructive comments on our manuscript entitled *“*Understanding challenges and enhancing the competency of healthcare providers for disability inclusive Sexual and Reproductive Health Services in rural Nepal.”

A revised version (clean and track changes) reflecting the point-by-point response to the reviewers’ comments have been submitted for your consideration. We believe that we have addressed all the comments and that the manuscript has significantly improved.

Should you require any additional information, please do not hesitate to let me know. Thank you very much for your time and kind considerations.

Your sincerely,

**Pabitra Neupane**

**RESPONSE TO THE REVIEWERS**

Journal Requirements:

When submitting your revision, we need you to address these additional requirements.

1. Please ensure that your manuscript meets PLOS ONE's style requirements, including those for file naming. The PLOS ONE style templates can be found at
   <https://journals.plos.org/plosone/s/file?id=wjVg/PLOSOne_formatting_sample_main_body.pdf> and 
   <https://journals.plos.org/plosone/s/file?id=ba62/PLOSOne_formatting_sample_title_authors_affiliations.pdf>

**RESPONSE:** All necessary adjustments have been made.

2. We note that your Data Availability Statement is currently as follows: All relevant data are within the manuscript and its supporting informations.

Please confirm at this time whether or not your submission contains all raw data required to replicate the results of your study. Authors must share the “minimal data set” for their submission. PLOS defines the minimal data set to consist of the data required to replicate all study findings reported in the article, as well as related metadata and methods (<https://journals.plos.org/plosone/s/data-availability#loc-minimal-data-set-definition>).

For example, authors should submit the following data:

- The values behind the means, standard deviations and other measures reported;
- The values used to build graphs;
- The points extracted from images for analysis.

Authors do not need to submit their entire data set if only a portion of the data was used in the reported study.

If your submission does not contain these data, please either upload them as Supporting Information files or deposit them to a stable, public repository and provide us with the relevant URLs, DOIs, or accession numbers. For a list of recommended repositories, please see <https://journals.plos.org/plosone/s/recommended-repositories>.

If there are ethical or legal restrictions on sharing a de-identified data set, please explain them in detail (e.g., data contain potentially sensitive information, data are owned by a third-party organization, etc.) and who has imposed them (e.g., an ethics committee). Please also provide contact information for a data access committee, ethics committee, or other institutional body to which data requests may be sent. If data are owned by a third party, please indicate how others may request data access.

**RESPONSE:** The codebook produced as the outcome of the data analysis, the interview guideline for the KII and FGDs and the informed consent forms have been available for this study. As participants have not consented for the available of full interview transcripts during this study, it has not been publicly available to respect participants anonymity and consent.

3. Please include your full ethics statement in the ‘Methods’ section of your manuscript file. In your statement, please include the full name of the IRB or ethics committee who approved or waived your study, as well as whether or not you obtained informed written or verbal consent. If consent was waived for your study, please include this information in your statement as well.

**RESPONSE:** This section has been updated in Methodology and it reads as: the data was collected from 10^th^ December 2023 to 15^th^ January 2024. Written ethical approval was obtained from the Ethical Review Board of Nepal Health Research Council (Ref. Number 783) before conducting the data collection. The research follows standard ethical principles guided by the Helsinki Principles. The autonomy and confidentiality of participants has been maintained throughout the project period. All the participants were oriented about the study and its importance. Prior to data collection, written consent was taken from the participants after orientation, verbal consent was taken before starting the interviews and their right to withdraw from the research was reiterated. The participants were assured of anonymity and confidentiality before, during and after the study.

4. We note you have included a table to which you do not refer in the text of your manuscript. Please ensure that you refer to Table 1 in your text; if accepted, production will need this reference to link the reader to the Table.

**RESPONSE:** This comment has been addressed in line number 167

5. Please include captions for your Supporting Information files at the end of your manuscript, and update any in-text citations to match accordingly. Please see our Supporting Information guidelines for more information: <http://journals.plos.org/plosone/s/supporting-information>.

**RESPONSE:** All the supporting files has been made available in the manuscript so this is not applicable for this study.

**Reviewer’s Comment**

**Reviewer #1:**

1) It would be useful for the reader to understand what the national guideline for disability-inclusive services in Nepal says about providing SRH services to women with disabilities. It is unclear whether there are already provisions, but the study is exploring the challenges to implementing these, or whether the guidelines are incomplete/insufficient and the challenges being explored in the study go beyond these.

**Response:** Thank you so much for your comment. The additional policy provision and mechanism has been adequately mentioned in the introduction section from line numbers 76-93. This comment has been addressed completely.

2) In the same vein, it would be important to clarify whether the authors have a specific understanding of what healthcare provider competency entails in this case, and whether they know what the services provided to women with disabilities ought to be.

**Response:** Thank you so much for your feedback. The specific understanding of what healthcare providers competency is expected has been discussed in discussion section from line number 440-447. This comment has been addressed completely.

3) The sub-topic on gender sensitiveness should be developed further to ensure it is well argued and supported. As it stands now, it is unclear how the conclusion that there is a gender bias in care is arrived at.

**Response:** We discussed on this as a team, and we agree that this study does not have enough evidences to establish lack of gender sensitiveness apart from one or two excerpts. Thus, we have removed 'gender sensitiveness' from the topic and sub-theme and removed 'gender bias' from conclusion to avoid any misleading information and discussion. It is discussed under discrimination but not as a separate sub-section. This comment has been addressed.

4) When describing the results on healthcare provider knowledge it would be important for the reader to know what they are expected to know. Overall, concluding that healthcare providers have limited knowledge and skills needs to be sustained by contrasting the findings with what they should know/the expected competencies they should hold. For instance, in the discussion the following claim is made: "Though there is inclusion of some clinical components of disability in the curriculum under the general practice subject, the contents are not adequate to understand and cater to the SRH needs of women or to provide disability inclusive SRH services to women". Although the authors may know what the adequate contents should be, the reader may not and it is important to make this explicit so that the reader can follow and draw their own conclusions. Many of these issues could be addressed by providing some context on healthcare for people living with disabilities drawn from documentary analysis (policy, guidelines).

**Response:** Thank you so much for your comment. The additional policy provision and mechanism has been adequately mentioned in the introduction and discussion section.

4) The text is intelligible and overall clear, although minor grammar mistakes can be found throughout and should be addressed - for example, the use of the term 'adopted' when it is clear what is meant is 'adapted'. The formulation of sentences should be revised to improve clarity. The manuscript should be revised and edited accordingly.

**Response:** The manuscript has been thoroughly revised and checked for any language and grammatical error. This comment has been addressed.

Reviewer #2:

 The authors should clarify the following points in the manuscript:
1. In Study design and setting: the authors should clarify how many municipalities and health institutions of the districts were covered by the study?

**Response:** The number of rural/municipalities and health facilities has been mentioned. However, the name of municipalities and health institutions has not been mentioned to ensure anonymity and confidentiality of health institutions and facilities. The socio-demographic information is in detail and naming the municipalities and health institutions could lead to participant identification.

2. In Line no. 125: For which level of health professional did the authors intend to refer by the term "health in-charge"?

**Response:** The term "health in-charge" has been corrected to "health facility in-charge" for clarity. The health facility in-charge are usually medical doctors, H.A, or senior AHW.

3. In Line no. 224: The authors mentioned the finding that there is a lack of equipments including CT and MRI for the disability services. Authors need to clarify the need of these highly valued equipment for SRH in rural setting for disability.

**Response:** The health facility under the study are health posts, Primary Health Care Centers and Hospitals at district/province level and the need for equipment, commodity and tool varies. The CT and MRI are required for abdominal or pelvic screening/assessment at district level while the need of health post include USG, fetal doppler, Pap-Smear test kit, Silicon Ring Pessary. This has been clarified in the discussion section.
