# Supplementary material for: Understanding challenges and enhancing the competency of healthcare providers for disability inclusive sexual and reproductive health services in rural Nepal
Source: PLoS One. 2024 Dec 13;19(12):e0311944. doi: 10.1371/journal.pone.0311944 (PMC11642985; doi:10.1371/journal.pone.0311944)
Supplement: Attachment — Submitted filename: Response to reviewers.docx [file pone.0311944.s003.docx]

**27^th^ September 2024**

**Yitagesu Habtu Aweke, Ph.D**

Academic Editor, PLOS ONE

**RE: Revision of Manuscript PONE-D-24-16726R2**

Dear Editor,

We would like to thank you and the reviewers for your time and providing constructive comments on our manuscript entitled *“*Understanding challenges and enhancing the competency of healthcare providers for disability inclusive Sexual and Reproductive Health Services in rural Nepal.”

We are thankful to reviewers for their encouraging words and praises related to manuscript and also identifying areas of improvement. A revised version (clean and track changes) reflecting the point-by-point response to the reviewers’ comments have been submitted for your consideration. We believe that we have addressed all the comments and that the manuscript has significantly improved and ready to get published.

Should you require any additional information, please do not hesitate to let me know. Thank you very much for your time and kind considerations.

Your sincerely,

**Pabitra Neupane**

[neupanepavitra@gmail.com](mailto:neupanepavitra@gmail.com)

**RESPONSE TO THE REVIEWERS**

**Journal Requirements:**
Please review your reference list to ensure that it is complete and correct. If you have cited papers that have been retracted, please include the rationale for doing so in the manuscript text or remove these references and replace them with relevant current references. Any changes to the reference list should be mentioned in the rebuttal letter that accompanies your revised manuscript. If you need to cite a retracted article, indicate the article’s retracted status in the References list and also include a citation and full reference for the retraction notice.

Response: The reference list has been checked and the reference is updated accordingly.

**Response Reviewer’s Comment**

Reviewer #3: The authors have successfully addressed the feedback provided by the previous reviewers, demonstrating commendable engagement with critical suggestions. Upon reviewing this revised submission, I recommend it for publication. This study is particularly notable for its focus on a critically underexplored area disability-inclusive sexual and reproductive health services in rural Nepal. The authors' examination of healthcare providers' competencies in delivering these services is highly relevant, especially given the persisting barriers faced by women with disabilities, including knowledge gaps in disability management, biased perceptions, and inadequate communication skills.

The use of a qualitative, thematic research design, supported by key informant interviews and focus group discussions, has allowed for an in-depth exploration of these complex issues. The findings, which highlight significant gaps in procedural skills and the need for disability-inclusive training and infrastructure, provide valuable insights for improving health systems in underserved regions.

Considering that the authors are university students, the depth and rigor of their work are commendable. They have contributed meaningfully to an area of research that has clear implications for both policy and practice in the context of rural health services in Nepal. Therefore, this manuscript contributes to the field and should be published.

**Response:** Thank you for your and encouraging feedback. We greatly appreciate your recognition of the importance of this research and are grateful for the opportunity to contribute to the underexplored area of disability-inclusive sexual and reproductive health services. We look forward to seeing our work published and hope it informs future policies and practices in rural health services.

**Reviewer #4: Abstract**

1. Result (Line 38-45): This sentence is long and hard to follow. It's important to clarify this for the reader to easily grasp the meaning.

**Response:** Thank you so much for your comment. The comment has been addressed in the manuscript in the line number 37-45. We have broken the single sentence into multiple for readability and clarity.

2. Conclusion (Line 46-50): Instead of writing one long sentence (which spans four lines), consider breaking it into simpler sentences to improve clarity.

**Response:** Thank you so much for your feedback. The comment has been addressed in the manuscript from line number 48-54. We have broken the single sentence into multiple simpler sentences for readability.

3. Introduction

- Line 90: There are two periods (dots).

**Response:** The comment has been addressed in the manuscript line number: 92. We have removed the extra dot.

4. Method (Lines 145-150):
   Participants and Sampling:

- The criteria for inclusion/exclusion need more details. Do you include both males and females? What is the age range? What languages do participants speak (Nepali or English)?

**Response:** Thank you so much for your comment. The additional information about the participants and inclusion has been added in the manuscript, line number: 132-135, 148-151

5. Results

- Line 176: Double-check the spacing between words.

**Response:** Thank you for the comment, we have double checked the spacing between words and there is no any error.

- Lines 208-210: This sentence is somewhat unclear. Are healthcare providers aware of advocacy?

**Response:** Thank you so much for the comment. This comment has been addressed in manuscript, line number 212

6. Discussion:
   • “Professionalism and Behavior of Health Care Providers”: This section presents clear points and is easier to follow compared to the previous section, “Disability-Inclusive Health System,” which I have already commented on for revision.

**Response:** Thank you for your positive remarks on this. We have tried to present the findings in the sections with clarity.

7. Line 428: There is an extra period (dot).

**Response:** The comment has been addressed in the manuscript line number: 433. We have removed extra dot.

We have thoroughly reviewed the manuscript for language edit.
